# Prevalence of mental disorders in French prisons for men

**DOI:** 10.1186/1471-244X-6-33

**Published:** 2006-08-21

**Authors:** Bruno Falissard, Jean-Yves Loze, Isabelle Gasquet, Anne Duburc, Christiane de Beaurepaire, Francis Fagnani, Frédéric Rouillon

**Affiliations:** 1 Inserm U669, Maison de Solenn, 97 Bd du Port Royal, 75679 Paris, France; 2 Université Paris-sud 11, 94000 Le Kremlin Bicêtre, France; 3 Département de santé publique, Hôpital Paul Brousse, APHP, 14 Av Paul Vaillant Couturier, 94804 Villejuif, France; 4 BRISTOL-MYERS SQUIBB, 3, rue Joseph Monier BP 325, 92506 Rueil-Malmaison, France; 5 CEMKA-EVAL, 92340 Bourg-la-Reine, France; 6 SMPR, Prison de Fresne, 94000 Fresne, France; 7 Univiversité Paris 5, 75015 Paris, France; 8 CMME, Hôpital Sainte-Anne, 75014 Paris, France

## Abstract

**Background:**

Psychiatric surveys conducted in prison populations find high prevalence rates, but diagnoses may be difficult in this particular context. None of these surveys have been conducted in France.

**Methods:**

800 incarcerated male were sampled at random. Each prisoner was interviewed by a group of 2 clinicians, at least one of them being a senior psychiatrist. One of the clinicians used a structured clinical interview which generated DSM IV diagnosis (MINI plus); the second completed the procedure with an open clinical interview.

**Results:**

Prevalence rates for a diagnosis given independently by both clinicians and for a consensual diagnosis were respectively: 3.8% (6.2%) for schizophrenia, 17.9% (24%) for major depressive disorder, 12.0% (17.7%) for generalized anxiety and 10.8% (14.6%) for drug dependence.

**Conclusion:**

Psychiatric diagnosis can be difficult to interpret in prison, especially using traditional standardized interviews. The approach proposed here, with good reliability and closer to a day-to-day clinical practice, yields high prevalence rates.

## Background

In western countries, many important and valuable psychiatric surveys have been conducted in prison populations. A systematic review of 62 surveys was reported in 2002 [1]; it found prevalence rates as high as 3.7% for psychotic illnesses and 10% for major depression.

Such results are however difficult to interpret. They are most often obtained from trained lay-interviewers who make their diagnoses from validated questionnaires such as the Diagnostic Interview Schedule or the Composite International Diagnostic Interview. But it is often suggested that these lay-administered interviews tend to underestimate prevalence as compared to clinician-administered interviews [2]; moreover the validity of these instruments in a population of prisoners is open to question [3]. Indeed, bereavement, stress and situations of de-realization are widespread in prison. It should be added that symptoms related to depression, anxiety and even psychosis need to be considered with caution and assessed by an experienced clinician.

About 65000 people were in prisons in France in January 2004 [4]. A few psychiatric surveys have been conducted in this population [5,6], but none has proposed reliable estimates of the prevalence rates of mental disorders. Indeed, these studies have dealt only with newly admitted prisoners, in non-random prison samples; furthermore, diagnoses were made using either a traditional clinical way [6] or a non-validated instrument [5].

In 2002, the French ministries of health and justice decided to estimate the prevalence of mental disorders in French prisons through an epidemiological study. These prevalence estimates were to be made from: 1. diagnoses based on DSM-IV definitions [7], 2. diagnoses with clinical significance guaranteed by the opinion of a senior psychiatrist and by a minimum threshold of severity, 3. diagnoses with a high degree of reliability

To achieve these objectives, the following methodology was used.

## Methods

### Population and sampling

There are 3 types of prison in France. The "*Maisons d'arrêt*" are intended for remand prisoners and/or for prisoners with short sentences. The "*Maisons centrales*" are intended for prisoners with long sentences, entailing maximum levels of security. The "*Centres de detention*" are intermediate.

Prisoners were chosen at random using a two-stage stratified random sampling strategy: 20 prisons were first selected at random from the list of all French metropolitan prisons for men with stratification on the type of prison ("*Maisons centrales*" and "*Centres de detention*" were over-represented, this has been taken into account in the statistical analysis); second, prisoners were chosen at random in each of these 20 prisons until 800 prisoners were enrolled. The sample size of 800 was chosen so that a prevalence of 10% had a 95% confidence interval of about [7.8%,12.2%] with a condition of statistical independence of each subject sampled. Since subjects sampled from the same prison cannot be considered independent in terms of psychopathology (in other words there is possibly a design effect), this confidence interval maybe wrong: for a design effect equal to 4, the previous confidence interval will enlarge to [5.7%,14,3%].

Acceptance of the study by prison authorities was generally very good. 57% of prisoners were available and agreed to the interview so that a sample of 1402 prisoners was contacted between September 2003 and July 2004, producing a total of 799 interviews. The level of participation was stable across prison types: 63% for the "*Maisons d'arrêt*" (remand prisoners and/or short sentences), 53% and 54% for the two types of prison intended for longer sentences (no difference between these percentages, p = 0.33, chi square test with 2 degrees of freedom). Some information that could explain why certain prisoners did not participate was collected: 1% of prisoners did not participate because a judge (legal authority) did not agree to such participation, 88% because the prisoner refused and 11% because the prisoner was not available.

### Data collection

Each prisoner was interviewed for approximately 2 hours by a group of 2 clinicians (clinical psychologist or psychiatrist), both of whom were present during the whole interview. At least one of these clinicians had to be a qualified psychiatrist (he/she will be referred as the "senior" member of the team); it was essential that neither of them belonged to the medical team of the prison. In the study, 17 psychiatrists were recruited as "senior" clinicians and 15 psychologists or residents in psychiatry as "junior" clinicians.

The interviews began with the collection of the signed informed consent of the prisoner. Diagnoses were then recorded according to a semi structured procedure validated in a previous study [8]: one of the clinician used a structured clinical interview which generates DSM IV diagnosis (MINI plus v 5.0 [9]); the second, more experienced, completed the procedure with an open clinical interview of about 20 minutes, intended to be more clinically relevant. The interview continued with the completion of various socio-demographic questions, including personal, family and judicial history. At the end of the interview, each clinician independently summarized his or her list of diagnoses and scored the Clinical Global Impression severity scale (CGIs, [10]); finally, they met and concluded with a consensus list of diagnoses and a CGIs score. It should be noted that the "junior" clinician who used the MINI did not know the formal MINI-generated diagnoses at the time when he or she made his or her own clinical diagnoses.

### Diagnoses

This method generates four series of diagnoses, corresponding 1. to patients for whom both clinicians independently made the same diagnosis (strictly speaking, the two clinicians are not independent since the interview is the same. For convenience, we shall nevertheless use this term in the rest of the paper), 2. patients for whom at least one clinician made the diagnosis, 3. the consensus diagnoses and 4. the MINI diagnoses.

### Statistical analysis

Statistical analyses were carried out with SAS 8.6, except for the estimation of prevalence which was performed with the "survey" package of R 2.0.1. [11]. These estimations take into account the stratified nature of the two-stage randomization process.

The protocol was formally approved by the Pitié Salpétrière Hospital Ethics Committee and by the "*Commission Nationale Informatique et Liberté*" (French Commission on individual freedom and data storage).

## Results

### Description of sample

The median age was 37 years old (quartiles interval 28 – 47). The median length of imprisonment was 9 months (quartiles interval 4 – 21). About half the prisoners (49%) were in prison for at least the second time. 28% of prisoners had seen a children's judge before the age of 18; 28% reported childhood ill-treatment; 29% mentioned that a close member of their family had been in prison at some time. Finally, 16% had a history of hospitalization for psychiatric reasons.

### Inter-rater agreement for psychiatric diagnoses

As explained above, each prisoner received diagnoses from two clinicians. It is thus possible to estimate measurement error from inter-rater disagreement for the total sample of interviews.

For the CGIs score, the weighted Cohen's Kappa is equal to 0.91. Cohen's Kappa is equal to 0.87 for major depressive disorders; 0.53 for manic/hypo-manic episode; 0.68 for bipolar disorders; 0.76 for panic disorder; 0.79 for agoraphobia; 0.78 for post traumatic stress disorder; 0.77 for generalized anxiety; 0.91 for alcohol dependence; 0.95 for drug dependence; 0.76 for psychotic disorders, 0.64 for schizophrenia. All these values correspond to a "good" or "excellent" agreement [12].

### Severity and prevalence of diagnoses

According to the consensual CGIs, 13.3% of subjects were rated as "Normal, not at all ill", 16.2% were rated as "Borderline mentally ill", 14,5% as "Mildly ill", 20.5 as "Moderately ill", 22.9% as "Markedly ill", 10.2% as "Severely ill" and 2.4% "Among the most extremely ill patients". About 22% of prisoners were notified to the medical team of the prison (with the agreement of the prisoner, patients already followed for psychiatric reasons were not notified).

Table 1 presents current prevalence estimates (at the time of interview) of DSM-IV psychiatric disorders among patients for whom the consensus CGIs score is at least equal to 5 ("Markedly ill", "Severely ill" or "Among the most extremely ill patients"). It should be noted that for certain DSM-IV diagnoses (Bipolar disorder is the main example), the patient may not necessarily present symptoms at the time of interview, a history of depressive and/or manic episodes is sufficient for the diagnosis.

**Table 1:** Prevalence estimates (with standard deviations) of DSMIV diagnoses.

		**Both clinicians**	**At least one**	**Consensus**	**MINI**
*Mood disorders*		21.4 (3.9)	30.4 (5.2)	28.0 (4.5)	28.6 (4.6)

	Major depressive disorder	17.9 (3.8)	26.1 (5.2)	24.0 (4.6)	22.9 (4.1)
	Dysthymic disorder	3.2 (1.2)	7.0 (2.0)	4.8 (1.5)	1.5 (0.5)
	Bipolar I or II disorder (lifetime)	2.0 (0.4)	3.9 (0.8)	3.1 (0.7)	1.3 (1.0)
	Manic/hypomanic episode	2.1 (0.6)	7.5 (2.5)	3.6 (1.0)	4.2 (4.2)

*Anxiety disorders*		21.2 (4.3)	31.4 (5.5)	29.4 (5.2)	24.0 (4.1)

	Panic disorder WaWA	3.7 (1.4)	6.7 (2.3)	5.1 (1.7)	3.9 (1.5)
	Agoraphobia WHoPD	6.6 (2.0)	12.0 (3.6)	10.0 (3.0)	10.8 (3.4)
	Social phobia	6.8 (1.7)	12.5 (3.0)	11.0 (2.6)	8.8 (2.1)
	Obsessive compulsive disorder	3.7 (1.2)	7.9 (2.9)	5.5 (2.0)	5.7 (1.8)
	Post traumatic stress disorder	9.7 (3.5)	15.8 (4.8)	14.2 (4.3)	6.6 (2.1)
	Generalized anxiety disorder	12.0 (2.1)	19.6 (3.0)	17.7 (2.7)	15.4 (2.1)

*Substance-Related dis. Disorders*		14.0 (2.8)	20.8 (3.4)	19.1 (3.3)	14.1 (2.6)

	Alcohol dependence	9.4 (1.9)	12.9 (2.4)	11.7 (2.3)	8.7 (1.7)
	Drug dependence	10.8 (2.5)	16.2 (2.7)	14.6 (2.6)	8.9 (2.0)

*Psychotic disorders*		12.1 (3.0)	19.2 (5.0)	17.0 (4.6)	17.3 (4.5)

	Schizophrenia	3.8 (1.0)	8.0 (2.6)	6.2 (1.8)	11.9 (4.0)
	Brief psychotic or Schizophreniform dis.	0	0.2 (0.2)	0.2 (0.2)	0.3 (0.2)
	Schizoaffective disorder	1.0 (0.4)	2.7 (1.1)	2.6 (1.1)	0.9 (0.4)
	Delusional disorder	2.4 (0.6)	6.3 (1.8)	5.3 (1.6)	0.3 (0.2)

*At least one disorder*		27.4 (4.5)	37.7 (5.1)	35.9 (5.0)	33.9 (4.8)

Patients have a consensus CGIs score at least equal to 5 ("Markedly ill", "Severely ill" or "Among the most extremely ill patients")

As mentioned above, four series of diagnoses are obtained. A "both clinicians" diagnosis corresponds to patients for whom both clinicians independently made the same diagnosis, an "at least one clinician" diagnosis corresponds to patients for whom at least one clinician made the diagnosis; there are also a series of consensus diagnoses and the MINI diagnoses.

For schizophrenia, this yields: 3.8% for the "both clinicians" diagnosis, 8.0% for the "at least one clinician" diagnosis, 6.2% for the consensus diagnosis and 11.9% for the diagnosis derived from the MINI. For major depressive disorder the results are respectively 17.9%, 26.1%, 24.0% and 22.9%. Obviously, the rates for "both clinicians" are lower than those for "at least one clinician", and the same is true for the "consensus" rates. However all MINI diagnoses rates are lower than the "consensus" rates except for schizophrenia and manic/hypomanic disorders.

For practical reasons (meetings with a judge or a lawyer, etc.) or because of refusal, only 57% of selected prisoners participated in the interview. It is important to discuss the possible influence of such missing data on the prevalence estimates reported here. Under a hypothesis of prevalence estimates twice as high in the 43% of prisoners that did not respond, the prevalence of a "both clinicians" major depressive disorders would rise from 17.9% to 26%, the prevalence of drug dependence and schizophrenia would rise from 10.8% to 15.4% and from 3.8% to 5.4% respectively. In a hypothesis of prevalence estimates twice as low in the group of non-participants, the prevalence of major depressive disorders would fall to 14%, the prevalence of drug dependence would fall to 8.5% and that of schizophrenia to 3%.

## Discussion

Prevalence estimates found in this study are higher than most of those already published in similar studies. One of the main discrepancies concerns depression: 17.9% of prisoners presented a major depressive disorder while a systematic review of 62 surveys in general prison populations in western countries [1] finds an average prevalence of 10%. Several explanations may be suggested for the differences observed: 1. the instruments used in the studies reviewed were different; 2. none of the studies was conducted in France; 3. the majority of the 62 studies reviewed were conducted more than ten years ago and prevalence of mental disorders in prison may have increased in western countries. In favor of a higher level of prevalence rates in France, a paper published in 2004 [5] reports data concerning 2302 new prisoners in French prisons; prevalence of depression as assessed by a clinician in a traditional clinical way on the basis of ICD 10 criteria was as high as 27.4%. Moreover, the general population prevalence rates of affective disorders are higher in France than in other European countries [13]. Another discrepancy concerns the prevalence rate of 14% for alcohol or drug dependency. A recent systematic review [14] finds prevalence rates as high as 18% to 30% for alcohol abuse and dependence and 10% to 48% for drug abuse and dependence. However, this review included studies who sampled prisoners within 3 months of arrival into prison, while the present study is a two-stage stratified random sample of all prisoners. It is likely that a longer stay in prison tends to lower the rate of alcohol or drug dependence. Anyway, this prevalence rate of 14% for alcohol or drug dependency in a situation where the availability of alcohol or drug is supposed to be limited is an indication of a very high lifetime prevalence of alcohol or drug problems in this population, as it is found in most studies [14].

As regards the diagnosis methodology used in the present study, it can be noted that the majority of prevalence rates derived from the MINI, once weighted by a clinical significance criterion, are close to prevalence rates obtained from the pair of clinicians. One of the most noticeable exceptions concerns psychotic disorders. Indeed, schizophrenia, schizoaffective disorder, delusional disorder, etc. are likely to be the most challenging diagnoses for psychiatric epidemiology; this is all the more true in the very particular context of prison, where de-realization, ideas of persecution and even delusions have to be carefully considered. The approach proposed here tries precisely to tackle this problem. It is definitely a "different method of assessment", as has been called for in the literature in the field of schizophrenia [15]. Its interest consists in: 1. a structural interview, to improve reliability; 2. an open clinical interview conducted by a senior psychiatrist to guarantee a certain level of clinical relevance; 3. a double assessment, because it stimulates the attention of the raters and because it helps to interpret results: patients who are thought by both clinicians independently to have the disorder are likely to be typical patients, while patients with a diagnosis only from one clinician are more likely to be borderline for the pathology; 4. an independent assessment of severity using the CGI severity scale, which can be used to support the clinical relevance of each diagnosis. It is important here to point out a limitation of this CGI score: the CGI severity scale is related to a patient, not to a particular disorder. Thus, if a patient has simultaneously a diagnosis of depression and a diagnosis of schizophrenia with a CGI severity score of 6, it is not possible to know if it is depression, schizophrenia, or the conjunction of both that makes the patient "severely ill".

There are many other limitations concerning this study. Among them are the low response rate and the fact that personality disorders are not assessed. For the low response rate, sensitivity analyses were conducted. They did not substantially challenge the results, this should however be considered with caution since sensitivity analyses give only indications and not hard statistical results. Concerning personality disorder assessment, the temperament and character dimensions as proposed by C.R. Cloninger [16] were evaluated and it was possible to diagnose antisocial personality disorder with the MINI. Results will be presented in a forthcoming paper.

The last and most important question concerns the practical consequences of such results. In legal terms, French law changed in 1994: it now distinguishes on the one hand psychiatric disorders that remove discernment (subjects may be declared non-responsible in this situation and are often hospitalized in a psychiatric department) and, on the other, psychiatric disorders that merely altered discernment (subjects remain punishable and can be incarcerated). It could be hypothesized that this change has increased the frequency of incarceration of prisoners with a mental disorder: the percentage of criminal offenders considered non-responsible for psychiatric reasons was 0.9% before this law (1992), 0.5% in 1994 and since then, it has slowly decreased (0.4% in 1996 and 0.25% in 1997 [17]).

In medical terms, if 27.4% of French male prisoners present a clinically significant psychiatric disorder (table 1), does this mean that they all need some form of medical care? This is a well known issue, relating to the definition of psychiatric disorders [18]: are such definitions intended to help clinicians in their choice of an optimal treatment? Are they categories of abnormal functioning? Are they educational tools? Are they a reimbursement threshold for medical costs? etc. It is not so easy to answer such questions. What can be said here is that 3.8% of prisoners were clearly suffering from schizophrenia and required appropriate treatment; 14.1% of prisoners presented drug or alcohol dependency; more than 1 in 5 of the remaining prisoners presented a level of distress that would lead immediately, in a general setting, to medical treatment. There is a collective obligation to give these issues more thought.

## Conclusion

Psychiatric diagnosis can be difficult to interpret in prison, especially using traditional standardized interviews. The approach proposed here, with good reliability and closer to a day-to-day clinical practice, confirms the high prevalence rates found in most of published surveys. The mere 3.8% found in schizophrenia indicates that these high prevalence rates do not correspond only to a particularly high level of distress inherent to imprisonment.

## Competing interests

The author(s) declare that they have no competing interests.

## Authors' contributions

BF wrote the paper, participated in the study design and coordination and in the statistical analysis, JYL participated in the study design and study completion, IG helped to draft the manuscript, AD participated in the study completion and coordination and in the statistical analysis, CdB participated in the study design and supervision, FF participated in the study coordination and supervision, FR participated in the study design and supervision. All authors read and approved the final manuscript.

## Pre-publication history

The pre-publication history for this paper can be accessed here:


